# Deep Sequencing-Based Transcriptome Profiling Reveals Avian Interferon-Stimulated Genes and Provides Comprehensive Insight into Newcastle Disease Virus-Induced Host Responses

**DOI:** 10.3390/v10040162

**Published:** 2018-03-30

**Authors:** Weiwei Liu, Xusheng Qiu, Cuiping Song, Yingjie Sun, Chunchun Meng, Ying Liao, Lei Tan, Zhuang Ding, Xiufan Liu, Chan Ding

**Affiliations:** 1 Shanghai Veterinary Research Institute, Chinese Academy of Agricultural Sciences, Shanghai 200241, China; liuweiwei@shvri.ac.cn (W.L.); xsqiu@shvri.ac.cn (X.Q.); scp@shvri.ac.cn (C.S.); sunyingjie@shvri.ac.cn (Y.S.); mengcc@shvri.ac.cn (C.M.); liaoying@shvri.ac.cn (Y.L.); tanlei@shvri.ac.cn (L.T.); 2 College of Veterinary Medicine, Jilin University, Changchun 130062, China; dingzhuang@jlu.edu.cn; 3 School of Veterinary Medicine, Yangzhou University, Yangzhou 225009, China; xfliu@yzu.edu.cn

**Keywords:** Newcastle disease virus, RNA-seq, CEF, chicken, IFN response, transcript

## Abstract

Newcastle disease virus (NDV) is an avian paramyxovirus that causes significant economic losses to the poultry industry worldwide, with variations in NDV pathogenicity due to the differences in virulence between strains. However, there is limited knowledge regarding the avian innate immune response to NDV infection. In this study, transcriptional profiles were obtained from chick embryo fibroblasts (CEFs) that were infected with the highly virulent NDV Herts/33 strain or the nonvirulent LaSota strain using RNA-seq. This yielded 8433 transcripts that were associated with NDV infection. This list of candidate genes was then further examined using Gene Ontology (GO) and Kyoto Encyclopedia of Genes and Genomes (KEGG) analyses. It showed a high enrichment in the areas of cellular components and metabolic processes, with the cellular components possibly being associated with NDV pathogenicity. Among these 8433 transcripts, 3616 transcripts associated with interferon-stimulated genes (ISGs) were obtained; these transcripts are involved in metabolic processes, including protein phosphorylation and protein modification. These results provide further insight into the identification of genes that are involved in NDV infection. The global survey of changes in gene expression performed herein provides new insights into the complicated molecular mechanisms underlying virus and host interactions and will enable the use of new strategies to protect chickens against this virus.

## 1. Introduction

Newcastle disease virus (NDV) infection presents as a serious respiratory disease and can lead to death in poultry. Newcastle disease (ND) is one of the most important infectious diseases of poultry [1,2]. It is distributed worldwide and it has the potential to cause large economic losses in the poultry industry. The first outbreaks of ND were reported in the mid-1920s in Java, Indonesia, and Newcastle-upon-Tyne, England, were reported [3]. Within a few years, ND had spread throughout the world and became endemic in many countries [4].

NDV is a member of the family *Paramyxoviridae* (genus *Avulavirus* in subfamily *Paramyxovirinae*) [5]. NDV contains a negative-sense, non-segmented single-stranded RNA genome that is approximately 15 kb and encodes six structural proteins, nucleoprotein (NP), phosphoprotein (P), matrix protein (M), fusion protein (F), hemagglutinin-neuraminidase (HN), and the large polymerase protein (L), in the 3′ to 5′ direction [6]. NDV has been known to infect at least 250 bird species through either experimental or natural routes [5]. NDV strain virulence has been associated with F protein cleavage, and its virulence is generally determined in one-day-old specific pathogen-free (SPF) chickens and categorized as highly virulent (velogenic), intermediate virulent (mesogenic), or nonvirulent (lentogenic), according to the Intracerebral Pathogenicity Index (ICPI) [2,3,5].

Innate immunity is the first line of defense against pathogen invasion. Upon viral infection, the innate immune system is activated in an antigen-independent fashion. It relies on the ability of the host to recognize pathogens through specific pattern recognition receptors (e.g., Toll-like receptors, RIG-I-like receptors, or NOD-like receptors) [7,8]. Next, downstream signaling pathways are sequentially activated, resulting in the production of type-I interferons (*IFNs*) and many other inflammatory cytokines, such as *TNFα*, *IL-1*, and *IL-18*. The IFN response is one of the most important innate immune responses against viral infection. Hundreds of IFN-stimulated genes *(ISGs)* are activated during this response and play an important role in antiviral activity [9,10].

Chicken IFN was discovered in 1957, and the first chicken *IFN* gene was characterized in 1994 [11,12]. The double-stranded RNA-activated protein kinase *(PKR*), a key chicken *ISG*, was identified in 2004 [10]. So far, multiple studies have shown *IFN* responses to be triggered upon NDV infection. *IFN-alpha* (*IFN-α*), *IFN-beta* (*IFN-β*), and *IFN-gamma* (*IFN-γ*) were upregulated in primary chick embryo fibroblasts (CEFs) upon NDV infection [13]. *IFN-γ* showed a higher expression in the bursa of Fabricius of chickens that were infected with the NDV velogenic strain and its expression did not affect NDV replication in vitro [14,15]. In LPS-activated chicken bone-derived marrow DCs (mature chicken BM-DCs), the velogenic NDV strain Chicken/Guangdong/GM/2014 (GM) and the lentogenic NDV strain LaSota were able to suppress the expression of *IFN-α* after 24 h of infection [16]. Recently, one study utilized microarray and RNA-seq analyses and identified chicken *ISGs* in CEFs that were treated with a chicken type I interferon (*IFN-α*) [17]. This generated the first database pertaining to chicken *IFN* responses. However, the current knowledge pertaining to avian *IFN* responses remains sparse, and the role of the *IFN* response in chickens that are infected with NDV remains unknown. Herein, *ISGs* that are related to NDV infection in CEFs treated with the NDV strains Herts/33 (highly virulent) or LaSota (nonvirulent) were identified by constructing mRNA profiles via RNA-sequencing (RNA-seq). These results will contribute to an understanding of differences in infectivity between different NDV strains and the role of the *IFN* responses during NDV infection. The information generated by this study will be beneficial to vaccine development and other control strategies.

## 2. Materials and Methods

### 2.1. Cell lines and Viral Propagation

Freshly isolated CEFs were obtained from 10-day-old SPF chicken embryos (Beijing Vital River Laboratory Animal Technology Co., Ltd., Beijing, China). The CEFs were maintained in Dulbecco’s modified Eagle’s medium (DMEM; Thermo Scientific, Waltham, MA, USA) supplemented with 10% fetal bovine serum (FBS; Gibco, Thermo Scientific, Waltham, MA, USA) at 37 °C with 5% CO_2_. Cells were cultured until a cell density of approximately 80% confluency was reached. NDV Herts/33 and LaSota strains were obtained from the China Institute of Veterinary Drug Control (Beijing, China), and were propagated in 9- to 11-day-old SPF embryos. For the LaSota strain, the infected allantoic fluid was harvested after 96 h; while for the Herts/33 strain, it was harvested after embryo death. Viral infectivity was determined based on the 50% tissue culture infective dose (TCID_50_) and the samples were stored at −80 °C until use.

### 2.2. Viral Infection

CEFs were seeded in T75 flasks and were cultured overnight. When the cell density reached approximately 80% confluence, the cells were infected with Herts/33 or LaSota at a MOI of 1 and were incubated at 37 °C with 5% CO_2_ for 1 h. Then, the growth medium was replaced with DMEM supplemented with 2% FBS and was incubated for 12 h before harvesting. There was a cytopathic effect (CPE) on CEFs, which confirmed NDV infection [18,19]. Uninfected cells were regarded as negative controls and the experiment was repeated with three biological replicates.

### 2.3. Total RNA Extraction

Total RNA was extracted from the infected and negative control CEFs using TRIzol (Invitrogen, San Diego, CA, USA), according to the manufacturer’s instructions. RNA degradation and purity were assessed by 1% agarose gel electrophoresis, and purity was assessed further, examined using a NanoPhotometer^®^ spectrophotometer (Implen, Westlake Village, CA, USA). RNA quantification was performed using a Qubit^®^ RNA Assay Kit and Qubit^®^ 2.0 Fluorometer (Life Technologies, San Diego, CA, USA). RNA integrity was assessed using a RNA Nano 6000 Assay Kit with a Bioanalyzer 2100 system (Agilent Technologies, Santa Clara, CA, USA).

### 2.4. RNA-Sequencing

Transcriptome library construction protocols were provided by Beijing Novogene Biotechnology Co., Ltd., Beijing, China, with a total of 3 μg RNA utilized per sample. First, ribosomal RNA was removed using an Epicentre Ribo-zero™ rRNA Removal kit (Epicentre, Madison, WI, USA). Sequencing libraries were subsequently generated using rRNA-depleted RNA with a NEBNext^®^ Ultra™ Directional RNA Library Prep kit for Illumina^®^ (NEB, Ipswich, MA, USA), according to the manufacturer’s recommendations. All of the treatments were conducted in triplicate. Paired-end sequencing was performed on an Illumina HiSeq2500 sequencer (Illumina, San Diego, CA, USA) with a read length of 125 nucleotides.

### 2.5. Transcriptome Assembly

Transcriptome assembly and annotation protocols were provided by Beijing Novogene Biotechnology Co., Ltd. in China. Low quality reads that the number of bases whose quality sQ ≤ 5 makes up over 50% of all reads, reads containing adaptors and reads containing poly-N > 10% were filtered out from the raw data using an in-house Perl script developed by Novogene Bioinformatics Institute (Beijing, China). Once the clean data was obtained, Q20, Q30, and GC content were calculated. All of the subsequent analyses were based on the clean data with high quality. Reference genome and gene model annotation files were downloaded from Ensembl (Gallus_gallus-5.0, http://www.ensembl.org/index.html). An index of the reference genome was built using Bowtie v2.0.6 and paired-end clean reads were aligned to the reference genome using TopHat v2.0.9. The mapped reads from each sample were assembled using both Scripture (beta2) and Cufflinks (v2.1.1) [20,21]. The distributions of reads for known genes were analyzed using HTSeq [22].

### 2.6. Coding Potential Analysis

Four coding potential analysis software packages, CNCI, phyloCSF with default parameters, CPC and Pfam searches with default parameters of −E 0.001 and −domE 0.001, were used to assess the transcript coding potential [23,24,25,26]. Transcripts that were predicted to have coding potential by all four tools were filtered out and used as the candidate set of mRNAs.

### 2.7. Expression Analysis

To quantify the gene expression levels in response to NDV infection, Cuffdiff (v2.1.1) was used to calculate the fragments per kilobase of gene model per million mapped reads (FPKM) for coding genes in each sample. Differential expressional analysis was also performed using Cuffdiff, with an adjusted *p* < 0.05 deemed differentially expressed. Cuffdiff provides statistical algorithms for determining differential expression within digital transcripts or gene expression data using a model that was based on a negative binomial distribution [20].

### 2.8. GO Enrichment and KEGG Pathway Analysis

Gene Ontology (GO) enrichment analysis of differentially expressed genes was implemented using GOseq in the R package (v3.3.2, The R Project for Statistical Computing, http://www.r-project.org/) [27], with gene length bias corrected and a *p* < 0.05 was considered to be significantly enriched. Kyoto Encyclopedia of Genes and Genomes (KEGG) pathway analysis was performed using the KOBAS software via a hypergeometric test. Identified GO terms and KEGG pathways with a corrected *p* < 0.05 were considered significantly enriched [28].

### 2.9. ISGs Induced by NDV Infection

All of the transcripts were queried against the Interferome database (v2.01; http://interferome.its.monash.edu.au) [29], which contains avian interferon-stimulated genes that were identified by microarray analysis and RNA-seq data from primary chick embryo fibroblasts treated with a chicken type I interferon (*IFN-α*) [17]. GO and KEGG analysis were performed as described above.

### 2.10. Validation of RNA-Seq Data by Quantitative Real-Time PCR (qPCR) Analysis

Total RNAs from CEFs that were infected with Herts/33 or LaSota for 12 h were used for qPCR analysis, with non-infected cells used as negative controls. A subset of eight unigenes with annotations from statistical analysis of RNA-seq was randomly selected for qPCR analysis. Briefly, cDNAs were synthesized using oligo (dT) and M-MLV Reverse Transcriptase (Promega, Madison, WI, USA), according to the manufacturer’s protocols. cDNA was diluted tenfold serial from 1 to 10^−6^ and primers efficiency was tested using standard curve to validate the 2^−∆∆*C*T^ method. Quantification was performed using a standard SYBR Green PCR Kit (Dongsheng Biotech, Guangzhou, Guangdong, China) and Bio-Rad CFX96 Touch™ Real-Time PCR Detection System. *GAPDH* was used as an endogenous control. The qPCR reaction was performed under the following conditions: 94 °C for 5 min, and then 40 cycles at 94 °C for 15 s, annealing at 59 °C for 15 s, and extension at 72 °C for 15 s. The primers for eight target genes (Table 1) were designed according to Illumina sequencing data by using Primer Premier 5 (Premier Biosoft, Palo Alto, CA, USA), with all reactions performed in triplicate. Gene expression was quantified relative to GAPDH expression using the 2^−∆∆*C*T^ method. Correlation coefficient between RNA-seq and qRT-PCR results was calculated using Graphpad Prism6 (v6.02, GraphPad Software, La Jolla, CA, USA).

## 3. Results

### 3.1. Identification of Coding Transcripts in NDV-Infected CEFs

Freshly isolated CEFs were cultured overnight and were infected with NDV Herts/33 or LaSota for 12 h. Total RNA was then extracted and sequencing libraries were constructed and used for deep sequencing. A total of 841,843,014 raw reads were produced using the Illumina HiSeq2500 platform (Table 2). After removing low-quality reads and reads with adaptor sequences, 808,024,840 clean reads were obtained (accounting for 121.21 Gb) (Table 2). We then queried the clean reads against the latest reference genome (Gallus_gallus-5.0, http://www.ensembl.org/index.html) and mapped using TopHat (http://tophat.cbcb.umd.edu/). Finally, total 249,144,644 control reads, 173,024,178 Herts/33 reads, and 206,403,083 LaSota reads were matched to either a unique location or multiple genome locations (Table 2). The average mapping ratios of the total reads were approximately 84.83% (control), 61.87% (Herts/33), and 75.94% (LaSota). Transcriptome assembly was performed using cufflinks (http://cufflinks.cbcb.umd.edu/). In total, 61,531,748 control reads, 61,512,546 Herts/33 reads, and 74,973,347 LaSota reads were distributed in protein coding regions, with analysis being performed using HTSeq (Table 2).

### 3.2. Global Changes in Expression in Response to NDV Infection

**Figure 1:**
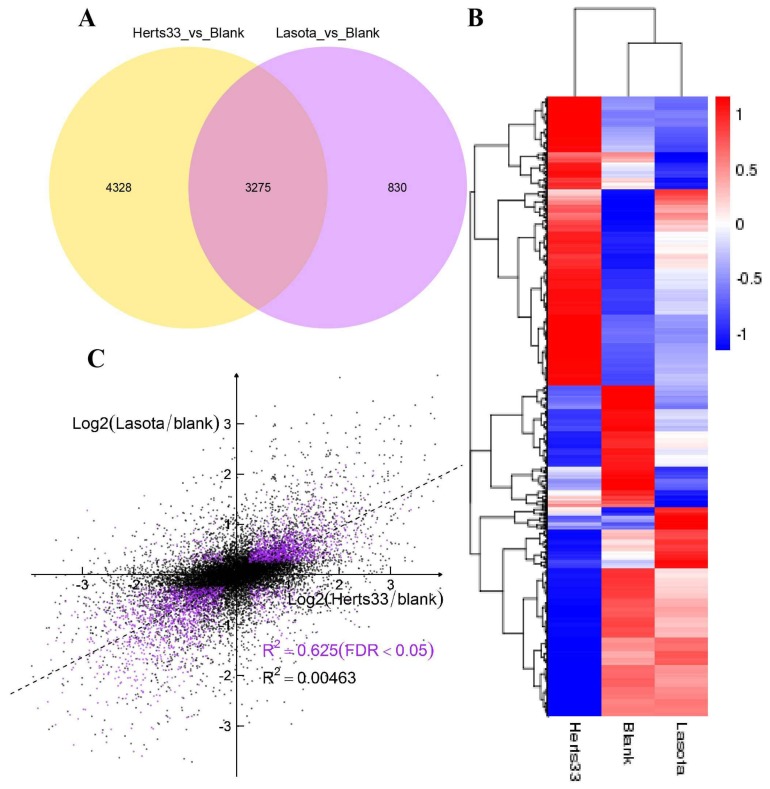
Summary of differentially expressed genes among the blank, Herts/33 and LaSota samples. (**A**) A Venn diagram of common differentially expressed genes when comparing two groups (blank vs. Herts/33 and blank vs. LaSota). (**B**) A hierarchical heat map showing transformed expressional values for the transcripts. Red indicates up-regulation and blue down-regulation. (**C**) Herts/33 and LaSota showed weakly correlated responses at the transcriptional level. Scatter plots reflect log2-transformed values for differential Herts/33 (x-axis) and LaSota (y-axis) expression relative to the blank control. All of the differentially expressed genes are indicated by black dots. Purple dots indicate differentially expressed genes with a cut-off threshold of false discovery rate (FDR) adjusted *p*-value < 0.05. The Spearman correlation coefficient (R) is also shown in purple.

Following the coding potential and the Cuffdiff analyses, a total of 8433 transcripts were identified in response to NDV infection. When comparing the control (blank) and Herts/33-infected CEFs (Herts/33_vs_blank), 7603 significantly differentially expressed transcripts were noted, with 3739 up-regulated (Log_2_Foldchange > 0) and 3864 down-regulated (Log_2_Foldchange < 0) (Supplementary Materials Table S1A). When comparing the LaSota-infected CEFs to the control (LaSota_vs_blank), 4105 transcripts were differentially expressed, with 1912 up-regulated (Log_2_Foldchange > 0) and 2193 down-regulated (Log_2_Foldchange < 0) (Supplementary Materials Table S1B). Differences in transcript abundances were then visualized via a Venn diagram and hierarchical clustering (Figure 1A,B).

The observed number of all genes associated with a Herts/33 infection was approximately two-fold greater than those observed during a LaSota infection. 3275 genes were common to both strains (Figure 1A, Supplementary Materials Table S1C). These findings indicate that there is a transcriptional difference between the infections by virulent and non-virulent strains. Additionally, when examining transcriptional correlations between Herts/33 and LaSota infections, the correlation coefficient was 0.00463. However, the correlation coefficient for the differentially expressed transcripts (*q* value < 0.05) between the Herts/33 and LaSota strains reached 0.625 (Figure 1C). These results indicate that there is a great difference between infection by virulent and non-virulent strains, with some changes in expression shared, regardless of virulence.

### 3.3. Functional Analysis of Differentially Expressed Transcripts

GO enrichment analysis was performed to classify the putative functions of differentially expressed transcripts when performing two-way library comparisons between the three groups (Control, Herts/33, and LaSota). Based on sequence homologies, the differentially expressed transcripts were separated into three main categories, which included cellular components, molecular functions, and biological processes.

Different gene functional distributions were noted upon comparing the transcriptional profiles of the CEFs that were infected by each of the viral strains to the control. When comparing Herts/33 to the control, the terms relating to metabolic processes were significantly enriched, including cellular, organic substrate, and macromolecular metabolic processes. The enriched terms that were associated with cellular components, such as cell parts, intracellular elements and organelle, and molecular functions, such as binding and protein binding, were significantly enriched. When comparing LaSota to the control, terms that were associated with developmental processes were significantly enriched. Cellular components related to cytoplasm and organelles were significantly enriched, with molecular functions associated with protein binding and ion binding significantly enriched.

**Figure 2:**
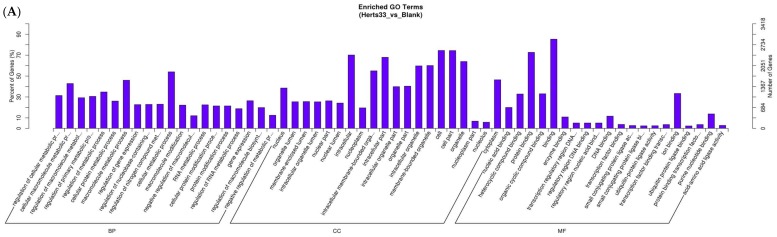

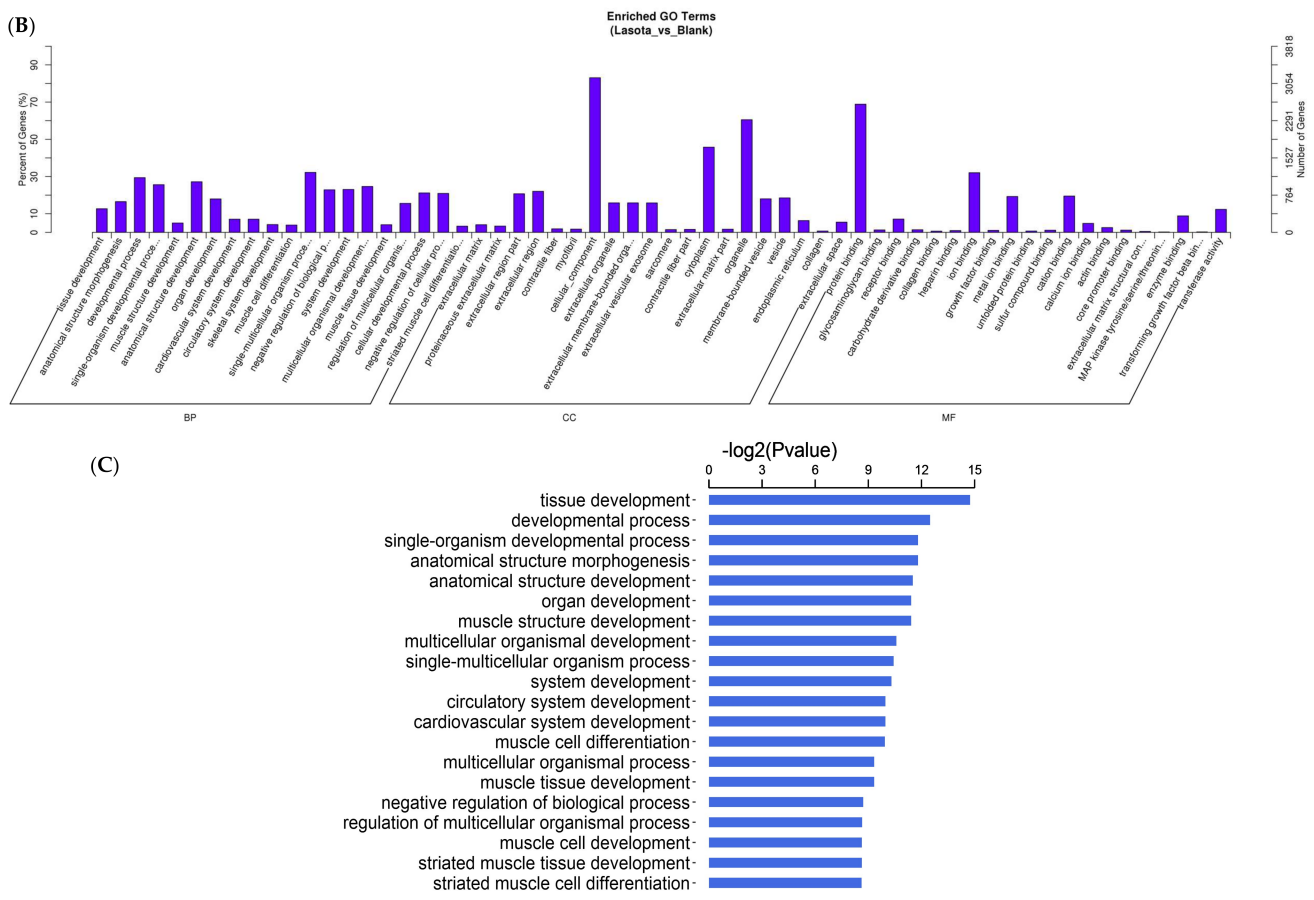
Gene ontology (GO) functional enrichment of differentially expressed genes. (**A**) GO analyses of differentially expressed genes in Herts/33 relative to the control; (**B**) GO analyses of differentially expressed genes in LaSota relative to the control; and, (**C**) GO analyses of differentially expressed genes common to both strains. Development processes were found to be the most enriched biological processes, judging by *p* value.

GO analysis of the genes that were differentially expressed in both strains, a total of 3275 genes, were examined based on their *p* values. The results revealed that most of the genes in common between both strains were enriched in the development process category, which is consistent with the terms that were obtained when comparing LaSota to the control because most of these genes are expressed during a LaSota infection (Figure 2C).

**Figure 3:**
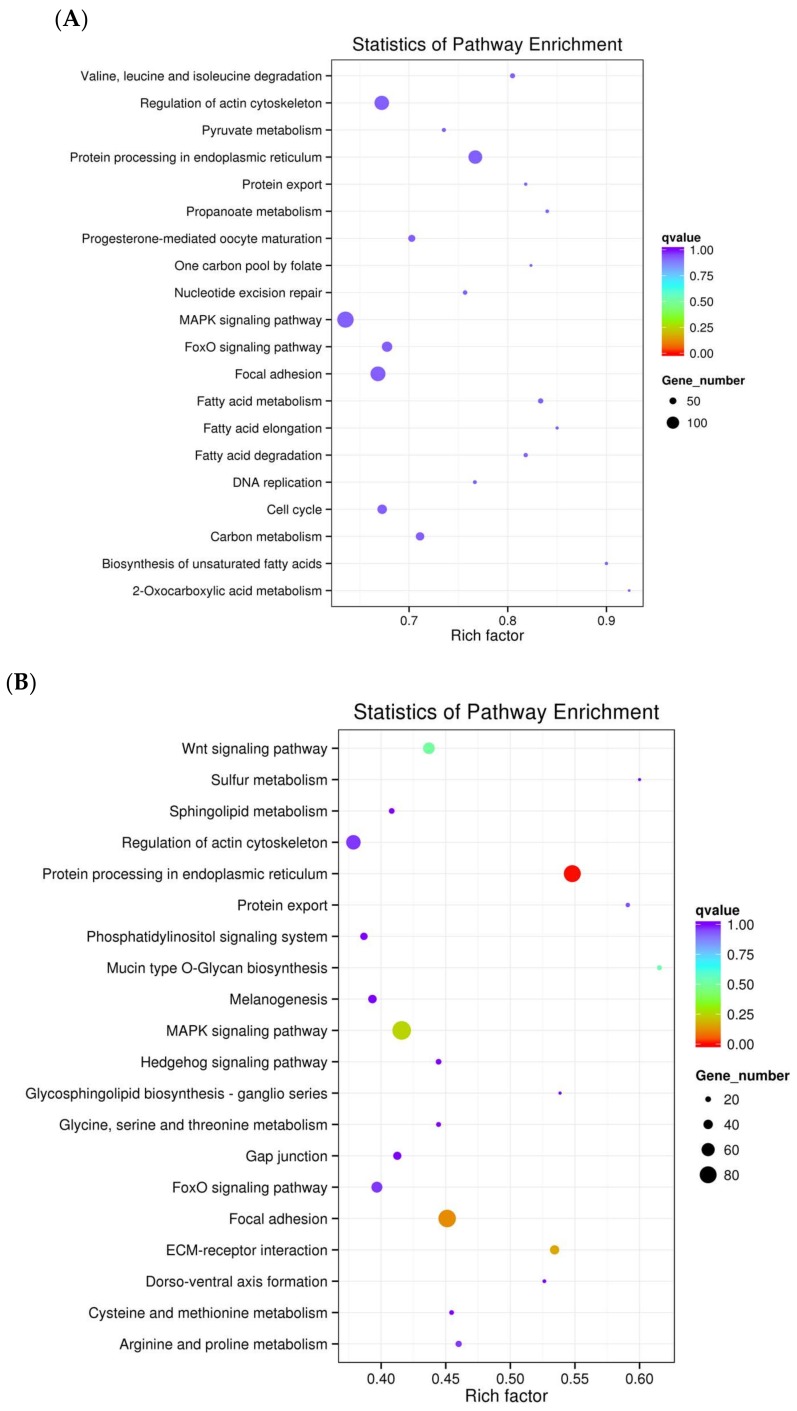
Kyoto Encyclopedia of Genes and Genomes (KEGG) annotation for differentially expressed genes. (**A**) KEGG analyses based on differentially expressed genes in Herts/33 relative to controls; and, (**B**) KEGG analyses based on differentially expressed genes in LaSota relative to controls. Circles indicate numbers of genes and colors depict the richness factor.

To further understand the functions of differentially expressed genes, the functions of the transcripts in Herts/33 and LaSota relative to the controls were mapped to pathways using the KEGG database. The functions of the transcripts were found to be mainly associated with protein processing in the endoplasmic reticulum (ER); fatty acid metabolism; regulation of actin cytoskeleton; valine, leucine, and isoleucine degradation; focal adhesion; and, carbon metabolism (Figure 3).

### 3.4. Analysis of Chicken ISGs in NDV-Infected CEFs

**Figure 4:**
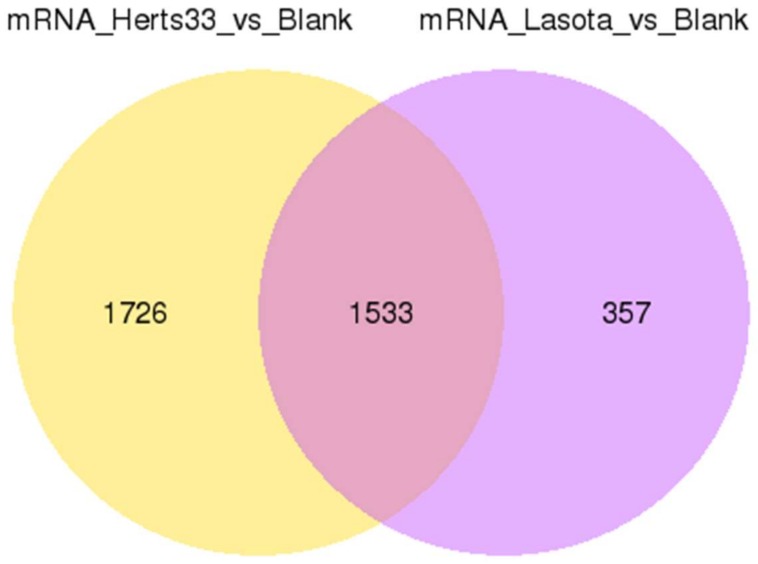
Summary of differentially expressed IFN-stimulated genes (*ISGs*) among the three samples. Venn diagram of common differential *ISGs* identified in comparison (blank vs. Herts/33 and blank vs. LaSota).

Innate immunity is the first line of defense against viral infection. Among the 8433 differential transcripts that were found to be associated with NDV infection, 3616 were associated with *ISGs*. When comparing Herts/33-infected CEFs to the control (Herts/33_vs_blank), 3259 significantly altered *ISGs* transcripts, comprising approximately 43% of the total genes in Herts/33 infection, were identified (Supplementary Materials Table S2A). When comparing the LaSota-infected CEFs to the control (LaSota_vs_blank), 1890 differential *ISGs* transcripts comprising 46% of total genes in the LaSota infection were identified (Supplementary Materials Table S2B). Transcriptional changes were further examined by constructing a Venn diagram (Figure 4).

**Figure 5:**
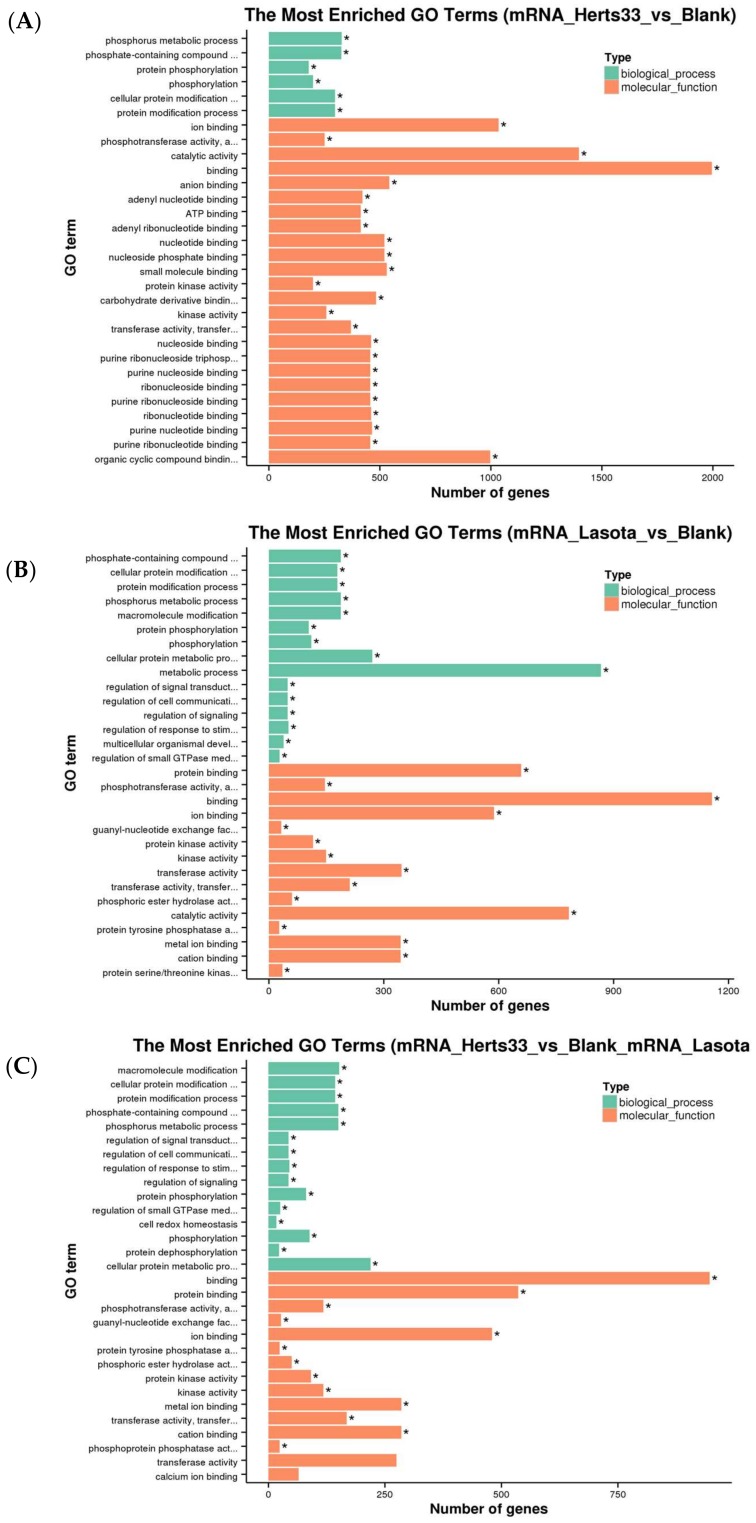
Gene ontology (GO) functional enrichment of differential *ISGs*. (**A**) GO analyses of differentially expressed *ISGs* in Herts/33 relative to the control (**B**) GO analyses of differentially expressed *ISGs* LaSota relative to the control; and, (**C**) GO analyses of differentially expressed *ISGs* common to both strains.

GO enrichment and KEGG pathway analyses were performed to classify the putative functions of the identified *ISGs* transcripts. When examining the differential *ISGs* in the Herts/33 group relative to the control, the most enriched biological processes were phosphorous metabolic process, phosphate containing compound, protein phosphorylation, phosphorylation, cellular protein modification, and protein modification process; with the most significant molecular function being binding (Figure 5A). For the differential *ISGs* in the LaSota group, the most significantly enriched biological process was the metabolic process; with the most significant molecular functions being binding (>1000) and catalytic activity (≈800; Figure 5B). Examination of the 1533 *ISGs* that were common to both of the strains showed them to be enriched in the categories of macromolecule modification, cellular protein modification, protein modification processes, phosphate-containing compounds, phosphorus metabolic processes, and cellular protein metabolic processes; with the most significant molecular functions being binding and protein binding (Supplementary Materials Table S2C and Figure S5C). KEGG pathway analysis showed that *ISGs* that were associated with NDV infection were mainly enriched in the regulation of the actin cytoskeleton, *MAPK* signaling pathway, protein processing in endoplasmic reticulum, and focal adhesion. These findings are relatively consistent with previous KEGG pathway analyses for the transcripts in the Herts/33 group relative to the control and LaSota group relative to the control.

### 3.5. Validation of RNA-Seq Data by Quantitative Real-Time PCR (qPCR)

**Figure 6:**
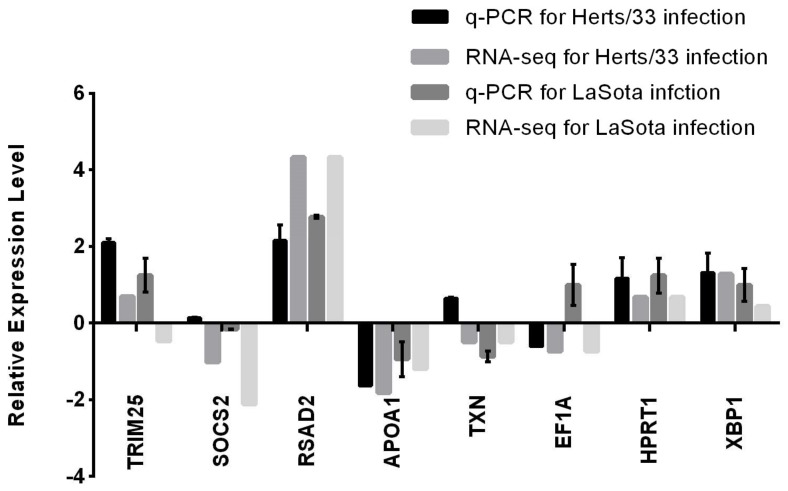
Verification of the relative expression levels quantitative real-time PCR (qPCR). Expression patterns of selected differentially expressed genes associated with NDV infection as determined by qPCR. The x-axis shows the annotations of the selected genes. The y-axis shows expression levels that are normalized to *GAPDH* expression.

To further validate the RNA-Seq data, a subset of eight unigenes with annotations from statistical analysis of RNA-seq was randomly selected for qPCR analysis. As shown in Figure 6, eight genes exhibited a concordant direction both in RNA-seq and qRT-PCR analysis. The correlation coefficient between RNA-seq and qRT-PCR results was 0.814 in Herts/33 infection and 0.800 in LaSota infection, respectively (Figure 6). These results support that the differential expression identified via RNA-Seq is reliable.

## 4. Discussion

This study involved a global transcriptome analysis of CEFs that were infected with two different strains of NDV. This approach enabled the determination of gene expression that was associated with NDV infection and the examination of candidate genes associated with an innate immune response in CEFs. In this way, this work provides new insights into host response to NDV infection and interactions between the virus and host and these findings will contribute to future studies.

With the development of next-generation sequencing, transcriptome analyses have been performed for many viruses, such as Zika, Epstein-Barr virus (EBV) and human cytomegalovirus (HCMV) [30,31,32]. Lung and trachea transcriptomes have also been obtained from resistant and susceptible chicken lines infected with NDV [33,34]. However, little is known regarding the cellular impact of NDV infection. Herein, RNA-seq, a next-generation sequencing method was combined with bioinformatics to analyze the CEF transcriptome following infection with the highly virulent NDV Herts/33 strain and the nonvirulent LaSota strain. In this study, many differentially expressed transcripts (>8000) associated with NDV infection were identified when compared with the few identified in previous studies [33,34]. This could be due to the use of velogenic in the current study or a different multiplicity of infection being used in other studies. Moreover, the higher number of identified transcripts during a Herts/33 infection relative to those that were identified during a LaSota infection could be attributed to differences in pathogenicity between the virulent and nonvirulent strains.

GO and KEGG pathway analyses were performed to classify the functions that were associated with the differentially expressed transcripts. These analyses showed that the biological processes that were enriched in Herts/33 infection were different from those enriched in LaSota infection. This finding suggests that infection with different viral strains could involve different biological processes. However, the exact mechanistic differences behind these observations would require further study.

Metabolism is the biological process involving a set of chemical reactions that modifies a molecule into another for storage, or for immediate use in another reaction, or as a by-product (Biology Online Dictionary, https://www.biology-online.org/dictionary/Metabolism). Studies indicate that viral infections require changes to host cellular metabolic networks [35,36]. In one study examining dengue virus (DENV) in primary human cells, central carbon metabolism, particularly glycolysis, was found to be necessary to support efficient viral replication [37]. Another study found that glutamine metabolism is essential to HCMV infection [38]. During an adenovirus E4ORF1 infection, anabolic glucose metabolism is promoted by virus-induced *MYC* activation and supports viral replication [39]. Previous studies showed NDV infection could affect DNA synthesis, thymidine metabolism, and vitamin metabolism [40,41,42]. In this study, GO and KEGG pathway analysis revealed that NDV infection also could resort to the host’s metabolic resources. The implicated metabolic pathways included fatty acid metabolism, amino acid metabolism and carbon metabolism. However, the specific molecule that enables NDV targeting of the host cell metabolism, and how it mechanistically functions to ensure its survival and replication, remains unclear.

In this study, 136 genes in Herts/33 infection and 89 genes in LaSota infection were found to be enriched from the mitogen-activated protein kinase *(MAPK)* signaling pathway, which enables the conversion of extracellular signals into cellular responses [43]. The *Raf/MEK/ERK* signal transduction cascade belongs to the *MAPK* cascade. *Raf/MEK/ERK* signaling leads to stimulus-specific changes in gene expression, alterations in cellular metabolism, and the induction of programmed cell death (apoptosis), thus modulating cellular differentiation and proliferation [44]. One study showed that NDV induces *NF-κB* activation in human renal carcinoma cells by activating the *p38 MAPK/NF-κB/IκBα* pathway. In clear cell renal cell carcinomas, the *p38 MAPK/NF-κB/IκBα* pathway was found to be involved in NDV infections and the subsequent induction of apoptosis [45]. Moreover, in A549/DDP cells, NDV-induced apoptosis occurred via the *caspase* pathway, while the *MAPK* and *Akt* pathways also contributed to apoptotic induction [46]. Additionally, *MAPK* pathways have been shown to be activated in NDV infected A549 cells, with *p38 MAPK* being involved in NDV-induced cell death [47].

Herein, genes that function in protein processing in the ER were obviously enriched. Accumulating amounts of evidence indicate that the ER is a crucial organelle that supports viral entry, replication, and assembly [48,49,50]. One study reported that a recombinant NDV LaSota strain (virulent) that was expressing the rabies virus glycoprotein (rL-RVG) could induce autophagy and apoptosis in gastric carcinoma cells by inducing ER stress [51]. The NDV NP and P proteins have also been found to induce autophagy via an ER stress-related unfolded protein response [52]. The NDV MTH-68/H strain was observed to selectively kill tumor cells by inducing ER stress and subsequent *p53*-independent apoptosis [53]. In this study, many genes that function in protein processing in the ER were found to be associated with NDV infection, but further examination is required to determine their roles in NDV-induced ER stress.

The *IFN*-coordinated response constitutes a major innate antiviral defense. However, although considerable research has been performed in humans and mice, little is known about avian *IFN* responses. When examining innate immunity in chickens that are infected with virulent NDV-CA02 in vivo, more than six thousand differentially expressed genes associated with innate immunity were identified using microarray, and a strong iNOS response was observed [54]. Recently, chicken *ISGs* were more broadly characterized using microarray and RNA-seq analyses [17]. Another study examined the differences in immune-related cytokine expression in velogenic versus lentogenic NDVs in infected chicken peripheral blood [55]. In a previous study, the NDV strains JS3/05 and JS5/05, which both have a VIId genotype, elicited stronger innate immune and cell death responses in chicken splenocytes than did the F48E8 or Herts/33 strains [56]. These studies demonstrated the NDV infection can cause strong innate responses. In this study, thousands of chicken *ISGs* and strong innate immune responses on NDV infection were also uncovered. These results provide an extensive list of *ISGs* that are associated with NDV infection and contribute to further understanding of the IFN responses in chickens.

In conclusion, this study presents chicken *ISGs* and the comprehensive insight into NDV- induced host responses and provides foundation for further studies that may elucidate the interactions between virus and host. Although this study has some limitations, such as the use of a single cell type in vitro and single time point, it nonetheless provides a comprehensive analysis of host transcriptional changes that occur during NDV infection and can provide new information for novel genes in chickens and a basis for future antiviral drug development. Future studies in other chicken cells investigating NDV infection will facilitate the validation of these findings and identify the specific mechanisms in gene regulatory networks of NDV-host interaction.

## Figures and Tables

**Table 1:** Primer sequences used for qPCR.

Gene Name	Forward Primer (5′–3′)	Reverse Primer (5′–3′)
*TRIM25*	TCAAGAGTCCCACCCTTCCA	AGCAGCTCAATGGACAGCAT
*SOSC2*	GCGCGCAGGGTGGTACT	ATGCGAACTGTCCCTAACCAA
*RSAD2*	ACACCTCAGGGAATCACCCTTT	AAGGATTCTCTGTTATCCAAGCTGAA
*APOA1*	GATGCCATCGCCCAGTTC	CCATGTCCTCACGCAGCTT
*TXN*	GTCTGTGTGACAAGTTTGGTGATG	AATGTTGGCATGCACTTCACAT
*EF1A*	GGGCACCTCATCTACAAATGC	ACCCAGGCGTATTTGAAGGA
*XBP1*	GTGCGAGTCTACGGATGTGAAG	CTGCAGAGGAACACGTAGTCTGA
*HPRT1*	CCAAACATTATGCAGACGATCTG	CCCATGCCCTTCATAATTTCA
*GAPDH*	CAATGATCCCTTCATCGATCTG	TTTCCCGTTCTCAGCCTTGA

**Table 2:** Summary of sequences analysis.

Category	Control_1	Control_2	Control_3	Herts/33_1	Herts/33_2	Herts/33_3	LaSota_1	LaSota_2	LaSota_3
Raw reads	109,126,046	110,981,726	86,062,988	83,496,140	94,400,454	93,150,904	93,479,188	83,743,856	87,401,712
Clean reads	104,687,082	106,667,980	81,878,214	80,208,914	90,581,186	89,441,660	89,772,806	80,667,246	84,119,752
Clean bases	15.7 G	16 G	12.28 G	12.03 G	13.59 G	13.42 G	13.47 G	12.1 G	12.62 G
Total mapped	89,050,776	91,232,554	68,861,314	52,421,734	59,372,721	61,229,723	72,781,410	65,496,022	68,125,651
	85.06%	85.53%	84.1%	65.36%	65.55%	68.46%	81.07%	81.19%	80.99%
Protein coding	32,050,717	32,705,773	25,620,258	18,519,665	21,280,793	21,712,088	26,453,027	24,048,464	24,471,856
	75.36%	75.45%	75.57%	74.08%	73.94%	73.54%	75.13%	75.16%	74.78%

Raw reads: all the original data produced by one sequencing; clean reads: reads remaining after removal of low quality reads and those reads with adapters or poly-N > 10%; clean bases: the sequence number multiplied by the length of the sequencing and converted to G units; total mapped: the number of reads that can be mapped to the genome; and protein coding: the distribution of reads in protein coding regions.

## Supplementary Materials

The following are available online at http://www.mdpi.com/1999-4915/10/4/162/s1, Table S1: Differentially expressed transcripts in Herts/33_vs_blank (Table S1A), LaSota_vs_blank (Table S1B) and common in both strains (Table S1C), Table S2: Differentially expressed ISGs in Herts/33_vs_blank (Table S2A), LaSota_vs_blank (Table S2B) and common in both strains (Table S2C).
